# Ammonia-oxidizing archaea and complete ammonia-oxidizing *Nitrospira* in water treatment systems

**DOI:** 10.1016/j.wroa.2022.100131

**Published:** 2022-03-14

**Authors:** Sarah Al-Ajeel, Emilie Spasov, Laura A. Sauder, Michelle M. McKnight, Josh D. Neufeld

**Affiliations:** Department of Biology, University of Waterloo, 200 University Avenue West, Waterloo, Ontario N2L 3G1, Canada

**Keywords:** Nitrification, Ammonia-oxidizing archaea, *Nitrospira*, Comammox, Ammonia oxidation, Water treatment, *amoA*

## Abstract

Nitrification, the oxidation of ammonia to nitrate via nitrite, is important for many engineered water treatment systems. The sequential steps of this respiratory process are carried out by distinct microbial guilds, including ammonia-oxidizing bacteria (AOB) and archaea (AOA), nitrite-oxidizing bacteria (NOB), and newly discovered members of the genus *Nitrospira* that conduct complete ammonia oxidation (comammox). Even though all of these nitrifiers have been identified within water treatment systems, their relative contributions to nitrogen cycling are poorly understood. Although AOA contribute to nitrification in many wastewater treatment plants, they are generally outnumbered by AOB. In contrast, AOA and comammox *Nitrospira* typically dominate relatively low ammonia environments such as drinking water treatment, tertiary wastewater treatment systems, and aquaculture/aquarium filtration. Studies that focus on the abundance of ammonia oxidizers may misconstrue the actual role that distinct nitrifying guilds play in a system. Understanding which ammonia oxidizers are active is useful for further optimization of engineered systems that rely on nitrifiers for ammonia removal. This review highlights known distributions of AOA and comammox *Nitrospira* in engineered water treatment systems and suggests future research directions that will help assess their contributions to nitrification and identify factors that influence their distributions and activity.

## Highlights

• AOA and comammox *Nitrospira* dominate low ammonia engineered environments.
• Ammonia and metal levels, along with temperature determine the niches of nitrifiers.
• Contributions to nitrification cannot be inferred solely by AOA or AOB abundances.
• Comammox *Nitrospira* may play an important role in engineered systems nitrification.
• Methods like SIP and FISH may help discern contributions from nitrifying guilds.

## Graphical abstract

**Figure:**
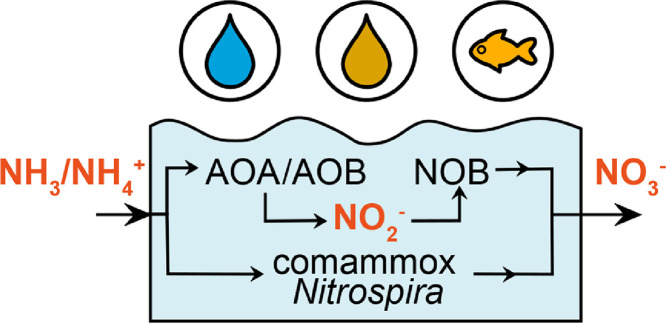

## Introduction

**Fig. 1:**
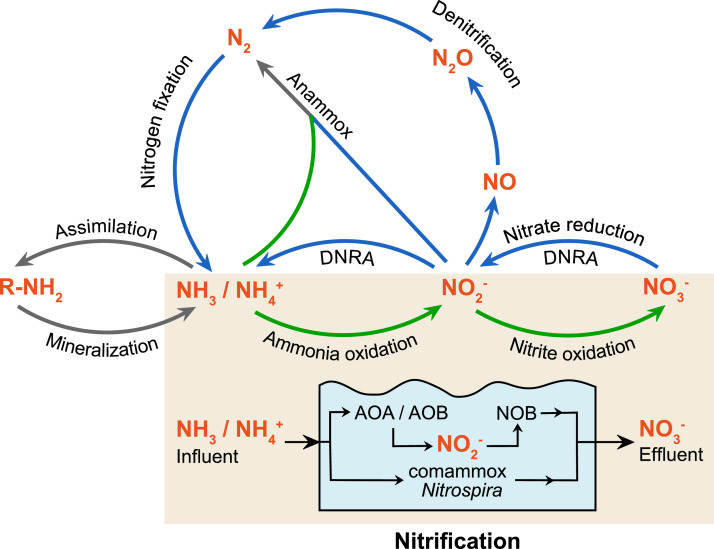
The nitrogen cycle, with an emphasis on nitrification-mediating microorganisms. Ammonia (NH_3_/NH_4_^+^) and nitrite (NO_2_^−^) oxidation are the first and second step of nitrification, respectively. Complete ammonia oxidation (comammox) involves both steps of nitrification within a single organism. Oxidation reactions are shown with green arrows, reduction reactions are shown with blue arrows, and non-redox reactions are shown with gray arrows. DNRA is dissimilatory nitrite reduction to ammonia, anammox is anaerobic ammonia oxidation. R-NH_2_ indicates organic molecules containing nitrogen as amine groups (i.e., biomass), NO is nitric oxide, NO_3_^−^ is nitrate, N_2_O is nitrous oxide, and N_2_ is dinitrogen. This figure is adapted from Stein and Klotz (2016).

Human activities produce domestic and commercial wastewater containing high concentrations of organic carbon and nitrogen, which is frequently treated to prevent negative impacts for receiving waters. An important part of this treatment is nitrification, which is the oxidation of ammonia to nitrate, a key step in the biogeochemical cycling of nitrogen (Fig. 1). Ammonia is toxic to aquatic life in relatively low concentrations, and although ammonia and nitrate both contribute to eutrophication in nitrogen-limited waters, nitrate is generally preferable because it has no direct oxygen demand, can be further converted to nitrogen gas by anaerobic respiration (e.g., denitrification and anammox), and has comparatively low toxicity for aquatic organisms.

**Fig. 2:**
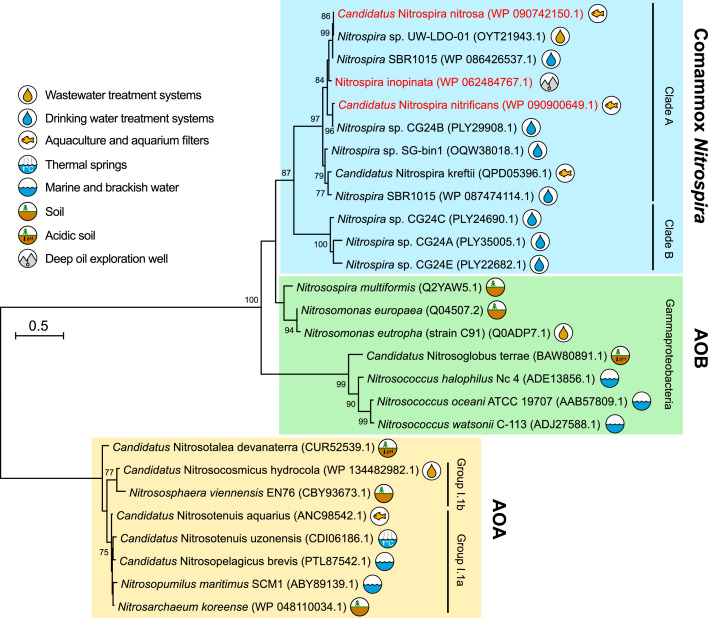
Phylogenetic relationships of AOA, AOB, and comammox *Nitrospira* based on AmoA amino acid sequences. Environments of origin for each sequence are grouped into major categories and shown to the right of each sequence entry. Comammox *Nitrospira* sequences originate from metagenomic and cultivation studies, with cultivated species shown in red. The sequence alignment is inferred using the Le Gascuel evolutionary model (Le and Gascuel, 2008), and the maximum likelihood method was used to construct the tree, using a discrete Gamma distribution to model evolutionary rate differences among sites (4 categories (+*G*, parameter = 2.9096)). The AmoA sequences of AOA were used to root the tree.

Nitrification is an important function within many natural and engineered environments, and involves two aerobic respiratory steps: ammonia oxidation and nitrite oxidation (Fig. 1). For over 100 years, only chemolithoautotrophic bacteria (ammonia-oxidizing bacteria, AOB) were thought to catalyze ammonia oxidation. These characterized nitrifiers belonged to the phylum *Proteobacteria*, with most species affiliated with the *Burkholderiales* (formerly *Betaproteobacteria*; i.e., *Nitrosomonas, Nitrosospira*), and others belonging to the *Chromatiales* (i.e., *Nitrosococcus*), all now classified within the class *Gammaproteobacteria* (Purkhold et al., 2000) (Fig. 2). First predicted by marine metagenomics (Treusch et al., 2005; Venter et al., 2004), and then demonstrated by a pure culture isolated from a marine aquarium (Könneke et al., 2005), members of the *Thermoproteota* (formerly *Thaumarchaeota*) can also oxidize ammonia (ammonia-oxidizing archaea, AOA). An anaerobic counterpart, anaerobic ammonia oxidation (anammox), is carried out by six bacterial genera associated with the *Planktomycetota* (formerly *Planctomycetes*) (Strous et al., 1999). The second step of nitrification, nitrite oxidation, is performed by nitrite-oxidizing bacteria (NOB) that are taxonomically diverse and belong to four different phyla (i.e., *Proteobacteria, Nitrospinota, Nitrospirota*, and *Chloroflexota*) (Daims et al., 2016).

Although the two steps of aerobic nitrification were long thought to be carried out by distinct microorganisms, theoretical predictions indicated that these steps could be conducted by individual microorganisms capable of complete ammonia oxidation (comammox), and that such microorganisms were likely to exist in the environment (Costa et al., 2006). Comammox bacteria were hypothesized to have high growth yields, low growth rates, and be competitive in biofilm habitats where ammonia mass transfer is low. A decade after the prediction of the existence of microorganisms mediating comammox, bacteria of the genus *Nitrospira* (Fig. 2) capable of carrying out both ammonia and nitrite oxidation were reported simultaneously by two research groups (Daims et al., 2015; van Kessel et al., 2015).

Many studies have explored the diversity, abundance, and activity of AOA in soil, marine, freshwater, estuary, and hot spring environments (Fig. 2). Given their recent discovery, less is known about the distribution and activity of comammox *Nitrospira*. Many engineered systems are specifically designed to reduce ammonia loads by promoting nitrification, such as domestic and industrial wastewater treatment plants (WWTPs), aquarium filters, aquaculture operations, and drinking water treatment systems. To enhance nitrogen removal and system operations, a more fundamental understanding of the community ecology and activity of ammonia oxidizers is essential. Exploring factors involved in niche differentiation of the three nitrifying guilds provides a basis for controlling nitrification. Recent reviews have focused on the presence and role of *Nitrospira* species in engineered systems (Mehrani et al., 2020), applied “biotechnological” potential of comammox (Lawson and Lücker, 2018), or the ecophysiology of comammox *Nitrospira* in various ecosystems (Koch et al., 2018). Given the relatively recent discoveries of AOA and comammox *Nitrospira* in engineered systems, this review focuses on summarizing recent literature examining the abundance, distribution, and activity of nitrifiers in water treatment systems.

### Ammonia-oxidizing archaea (AOA)

The AOA are broadly distributed in soil (Tourna et al., 2011), marine (Qin et al., 2014), and freshwater (French et al., 2012) environments, in addition to WWTPs (Li et al., 2016; Sauder et al., 2017). AOA can be classified into five main clusters based on phylogeny of the *amoA* gene, which encodes the active subunit of the ammonia monooxygenase (AMO) enzyme that catalyzes the first step of ammonia oxidation (Pester et al., 2012). The main clusters broadly distribute within Group I.1a “marine group”, which includes *Nitrosopumilus maritimus* (Könneke et al., 2005), and Group I.1b, “soil group”, which includes *Nitrososphaera viennensis* (Tourna et al., 2011) (Fig. 2). All cultivated AOA are thus far considered chemolithoautotrophs because they gain energy from ammonia oxidation and fix carbon dioxide into biomass (e.g., Könneke et al., 2014; Tourna et al., 2011; Walker et al., 2010). Previously, AOA were considered to have a higher affinity for ammonia than AOB (Martens-Habbena et al., 2009), which confers an advantage in ammonia-limited environments, but recent evidence suggests that cell surface area-to-volume ratios explain observed variations in affinity (Jung et al., 2021). Indeed, such high ratios could help explain a dominance of some AOA (and potentially comammox *Nitrospira*) in low ammonia environments, such as oligotrophic waters. The reported affinities of AOA for ammonia vary widely depending on species, with NH_3_ half-saturation constant (K_m_) values from ∼ 3 nM for *Nitrosopumilus maritimus* (Martens-Habbena et al., 2009) to 4.4 µM for *Candidatus* Nitrosotenuis uzonensis (Kits et al., 2017). In comparison, AOB typically have K_m_ values of 6 to 11 µM, but oligotrophic AOB have been reported to have NH_3_ K_m_ values in the range of 0.3 to 4.0 µM, putting them in a similar range as known AOA (Hu and He, 2017; Kits et al., 2017; Lehtovirta-Morley, 2018; Prosser and Nicol, 2012). This complicates a view of niche partitioning of ammonia oxidizing microorganisms based on ammonia affinity alone.

### Complete ammonia-oxidizing bacteria (comammox bacteria)

Existing enrichment cultures of comammox *Nitrospira* originate from engineered environments, including the trickling filter of an aquaculture system and a hot water pipe from a deep oil exploration well (Daims et al., 2015; Sakoula et al., 2021a; van Kessel et al., 2015). These aquatic habitats provide a surface for attached growth and are relatively oligotrophic, consistent with the predicted niche for comammox *Nitrospira* (Costa et al., 2006). Subsequently, comammox *Nitrospira* were discovered via metagenomics in a drinking water treatment plant, again processing water with relatively low ammonia concentrations (Pinto et al., 2015). Since the initial discovery of comammox, *Nitrospira inopinata* has been isolated in pure culture, facilitating study of its growth kinetics (Kits et al., 2017). Known comammox bacteria belong to *Nitrospira* lineage II (Daims et al., 2015; van Kessel et al., 2015), which is widespread in diverse marine, freshwater, soil, geothermal, and engineered environments (Daims et al., 2016, 2001).

In addition to enzymes that carry out nitrite oxidization, such as nitrite oxidoreductase (NXR), comammox *Nitrospira* genomes encode genes for enzymes that catalyze ammonia oxidation, including AMO and hydroxylamine dehydrogenase (HAO). The AMO-encoding genes of comammox *Nitrospira* are phylogenetically distinct from those of AOA and AOB, although they cluster more closely with AOB (Daims et al., 2015; Pinto et al., 2015; van Kessel et al., 2015; Fig. 2). Based on analysis of *amoA* genes affiliated with lineage II *Nitrospira*, Daims et al. (2015) identified two distinct clades of comammox *Nitrospira*: clades A and B. All existing cultivated species of comammox *Nitrospira* belong to clade A (Fig. 2). Among known *Nitrospira*, only comammox *Nitrospira* encode for ammonia oxidation genes, and therefore identification of comammox *Nitrospira* can be established through phylogenetic analysis of the *amoA* gene (Fowler et al., 2018; Palomo et al., 2018; Pjevac et al., 2017). All cultivated comammox *Nitrospira* are considered chemolithotrophs because they gain energy from complete ammonia oxidation to nitrate (Daims et al., 2015; van Kessel et al., 2015). Recent studies examined substrate affinity and kinetics of *N. inopinata* (pure culture) and *Ca.* N. kreftii (a highly enriched culture), revealing that both species have high apparent ammonia affinities (reported K_m(app)_ of 49–83 nM and 36 nM NH_3_, respectively), especially compared to AOB (Kits et al., 2017; Sakoula et al., 2021a). Only the marine AOA representative *N. maritimus* has a higher reported ammonia affinity than *N. inopinata* (Kits et al., 2017). As mentioned earlier for AOA, it is interesting to speculate that the surface-area-to-volume ratio may also account for the high substrate affinity exhibited by comammox *Nitrospira*. However, given the known gradient of ammonia affinities for AOA and AOB (Hu and He, 2017; Kits et al., 2017), and with only one pure culture of comammox *Nitrospira* with which to study affinity, it is premature to conclude that this characteristic of high ammonia affinity is broadly applicable to all comammox *Nitrospira*. As more comammox *Nitrospira* strains become available in pure culture, their kinetic characterization will help clarify why comammox *Nitrospira* appear to have a higher substrate affinity and how that impacts niche differentiation.

## AOA and comammox *Nitrospira* in wastewater treatment systems

Despite the importance of nitrifying microorganisms in wastewater treatment, the relative contributions of AOA, comammox *Nitrospira*, and AOB to wastewater nitrification remain unclear. In general, wastewater contains relatively high concentrations of ammonia, which should favor AOB over AOA and comammox *Nitrospira* (Costa et al., 2006; Martens-Habbena et al., 2009; Schleper, 2010). Indeed, many studies have reported the numerical dominance of AOB in municipal and industrial WWTPs (e.g., Mussmann et al., 2011; Wells et al., 2009; Zheng et al., 2021) and a high abundance of comammox *Nitrospira* have been detected in tertiary treatment systems, which have relatively low ammonia concentrations (Spasov et al., 2020).

Wastewater AOA were first identified based on archaeal *amoA* genes in DNA extracts from activated sludge of five municipal WWTPs, and sequencing revealed that the majority of these AOA belonged to the Group I.1b (Zhang et al., 2009). Subsequent studies demonstrated that AOA communities vary across WWTPs because of differing operational parameters (Oishi et al., 2012; Zhang et al., 2009). Another study sampled a single municipal WWTP weekly for one year and found that AOA were present in only ∼15% of the samples and were approximately 1000-fold less abundant than AOB (Wells et al., 2009). Several studies have corroborated these findings and demonstrated numerical dominance of AOB; for example, a survey of 52 industrial and municipal WWTPs in Europe found that AOB were numerically dominant in all but four WWTPs (Mussmann et al., 2011). In additional studies of various types of WWTPs in Beijing, both AOA and AOB were detected in a majority of samples, but AOB were numerically dominant in both municipal and industrial WWTPs (Gao et al., 2013, 2014). Additionally, AOB were reported to outnumber AOA in municipal WWTPs (Fan et al., 2017; Zheng et al., 2021), WWTP effluent (Huo et al., 2017), a WWTP treating landfill leachate (Yapsakli et al., 2011), and nitrifying bioreactors (Gao et al., 2016). The authors indicate that variable operating parameters such as temperature, dissolved oxygen concentration, short solids retention time, sensitivity to toxic compounds, and nitrate concentration as a possible explanation for the observed high AOB abundance, although no single factor appeared to govern the coexistence of AOA and AOB in WWTPs.

Despite many studies showing numerical dominance of AOB in WWTPs, some have found approximately equal abundances of AOA and AOB (Limpiyakorn et al., 2011; Reddy et al., 2014; Sonthiphand and Limpiyakorn, 2011), or reported AOA as numerically dominant (Limpiyakorn et al., 2011; Pan et al., 2018a; Roy et al., 2017; Sauder et al., 2017). A survey of six municipal and industrial WWTPs determined that, although both AOA and AOB were present in all samples, AOB dominated industrial coking and dyeing treatment plants, whereas AOA were more abundant in municipal WWTPs (Bai et al., 2012). In several municipal WWTPs, Group I.1b AOA outnumbered AOB (Chen et al., 2017; Pornkulwat et al., 2018; Sauder et al., 2017), demonstrating that municipal WWTPs are not exclusively dominated by AOB. Moreover, Pornkulwat et al. (2018) used DNA stable-isotope probing (DNA-SIP) to demonstrate that both AOB and AOA incorporated ^13^C-labelled bicarbonate in the presence of ammonia, suggesting that both AOB and AOA grow chemolithoautotrophically within the studied WWTP. Several studies have investigated the influence of ammonia on the relative abundance of AOA and AOB in WWTPs and suggested that ammonia concentrations are an important factor for shaping ammonia-oxidizing communities. When quantifying AOA and AOB *amoA* genes in sludge samples, incubation with high ammonia concentrations repressed transcription of archaeal, but not bacterial *amoA* genes (Fukushima et al., 2012; Limpiyakorn et al., 2011). Another study obtained municipal WWTP sludge with approximately equal numbers of AOA and AOB and demonstrated that, upon incubation with high ammonia concentrations, AOA abundances declined, whereas AOB population sizes were stable (Sonthiphand and Limpiyakorn, 2011). In the rotating biological contactors (RBCs) of a municipal WWTP, relative AOA abundances increased as ammonia concentrations decreased, suggesting a low-ammonia niche for these AOA (Sauder et al., 2012).

Although ammonia concentrations appear to influence ammonia-oxidizing community composition, additional factors are likely important. For example, temperature may play a role in the nitrifying communities of wastewater treatment systems. Studies have demonstrated that AOA were the dominant active ammonia oxidizers in WWTPs during cold months, as measured by labelled heavy carbon incorporation during autotrophic ammonia oxidation (Fan et al., 2018; Pan et al., 2018b). Fan et al. (2018) found that more AOA *amoA* genes were labelled than those of AOB, despite AOB outnumbering AOA in seed sludge. The active AOA were identified as *Ca.* Nitrosocosmicus hydrocola (formerly *Ca.* Nitrosocosmicus exaquare) and *Ca.* Nitrososphaera evergladensis. The authors suggest that AOA may be adapted to lower temperatures and may play a greater role in ammonia oxidation in WWTPs during the winter months than AOB. In addition to substrate affinity and temperature, exposure to varying levels of organic matter and metals, such as copper, may be an important factor involved in niche partitioning between AOA and AOB (Cao et al., 2011). Previous work has shown that several AOA and AOB species are inhibited by various concentrations of organic compounds (Clark and Schmidt, 1967; Jung et al., 2018; Lehtovirta-Morley et al., 2014; Qin et al., 2017). Recently, Gwak et al. (2020) revealed that several AOA and AOB strains present in WWTPs have varying sensitivities to organic compounds with high metal chelation potentials. This study demonstrated that organic compounds, like peptone and humic acids, sequester copper, thereby reducing availability to AOA for the production of copper-containing enzymes, including ammonia monooxygenase. As such, the addition of copper could reverse the inhibitory effects of organic compounds on AOA. Therefore, Gwak et al. (2020) concluded that the reduction of copper bioavailability by metal-complexing organic compounds may account for the lower abundance of AOA often observed in municipal WWTPs. Another study examined activated sludge from a WWTP deep oxidation ditch with high levels of bioavailable copper and determined that AOA were dominant ammonia oxidizers, based on both metagenomic and metatranscriptomic data (Yang et al., 2021). More recently, Koike et al. (2022) demonstrated that comammox *Nitrospira* in a groundwater fed bioreactor were also stimulated by copper supplementation, ultimately resulting in enhanced ammonia removal efficiency. The dominance of AOA and comammox *Nitrospira* over AOB in the activated sludge and groundwater fed bioreactors was explained by high copper and ammonia levels, and low oxygen concentrations in the system. Taken together, these findings suggest that AOA are relatively more dependent on copper and that this metal may play a role in niche differentiation between AOA and AOB.

Comammox *Nitrospira* were first discovered in WWTPs through screens of public database metagenomes (Daims et al., 2015; van Kessel et al., 2015), and multiple *amoA* amplicon sequence variants (ASVs) of clade A comammox *Nitrospira* were found in the same WWTP (Pjevac et al., 2017). Although these clade A comammox *Nitrospira* were less abundant than AOB, a substantial proportion (14–34%) of the detected *amoA* genes were affiliated with comammox *Nitrospira*. In both activated sludge and the biofilm of another WWTP, *amoA* genes similar to those of *N. inopinata* were found at low relative abundance (Chao et al., 2016). A one-year study of an activated sludge WWTP system detected comammox *Nitrospira amoA* genes at lower abundances than AOB and AOA *amoA* (Fan et al., 2017). Similarly, comammox *Nitrospira* were not found at high abundance in WWTPs in cold months (Pan et al., 2018b). However, it is important to note that these latter two studies used quantitative PCR (qPCR) primers to specifically target *amoA* genes of *N. inopinata*, and not all comammox-associated *amoA* genes, thus these surveys may not have quantified all comammox *Nitrospira* present. In contrast, Wang et al. (2018) found that comammox *Nitrospira* were more abundant than AOB in activated sludge of eight full-scale WWTPs. Comammox *Nitrospira* have also been found ubiquitously in a study of full scale biological nutrient removal WWTPs (Annavajhala et al., 2018), demonstrating that comammox *Nitrospira* could play a role in WWTP nitrification. Most studies have only detected clade A comammox *Nitrospira* in WWTPs and only one study reported clade B comammox *Nitrospira* populations in a WWTP (Xia et al., 2018).

Comammox *Nitrospira* were dominant nitrifiers in the microaerobic stage of a lab scale biological nutrient removal reactor (Camejo et al., 2017). With few AOA or AOB present, most ammonia oxidation was likely due to the detected *Nitrospira*. The comammox *Nitrospira* enriched from this reactor were classified as *Ca.* Nitrospira nitrosa. Additional studies reported that most comammox species detected in activated sludge samples from full-scale WWTPs and bioreactors with low dissolved oxygen levels were also closely related to *Ca.* N. nitrosa (Keene et al., 2017; Wang et al., 2018; Zhao et al., 2019). Furthermore, one study has found that comammox *amoA* transcript abundance either exceeded or was approximately equivalent to that of AOB in six out of eight WWTPs studied (Zheng et al., 2019). Most of these sequences clustered with *Ca.* N. nitrosa, and the authors suggest that comammox *Nitrospira* in this cluster could be the dominant group of comammox *Nitrospira* found in WWTPs with relatively high ammonia concentrations, contradicting earlier assumptions of adaptation to a low ammonia niche.

Comammox *Nitrospira* have been detected in several parts of WWTPs, including activated sludge aeration basins where ammonia concentrations are relatively high, as well as RBCs used for tertiary treatment where ammonia concentration is relatively low. A metagenomic study of the biofilm microbial community of RBCs found that comammox *Nitrospira* were the dominant ammonia oxidizers present (Spasov et al., 2020). Several *Nitrospira* metagenome-assembled genomes (MAGs) encoded genes for both ammonia and nitrite oxidation, showing that they were genetically capable of complete ammonia oxidation. It appears that many novel comammox *Nitrospira* populations co-existed in these RBCs. Prior work used CARD-MAR-FISH on RBC biofilm samples incubated with ammonia and labelled bicarbonate to reveal MAR-positive *Nitrospira* (Sauder et al., 2017). If many of these *Nitrospira* were comammox bacteria, as demonstrated by a subsequent study of this same system (Spasov et al., 2020), these findings likely implicate comammox *Nitrospira* in actively fixing bicarbonate while performing comammox reactions. Taken together, the high relative abundance and genetic diversity of those comammox *Nitrospira* ecotypes make them ideal targets for ongoing cultivation efforts.

It is important to note that from the context of ammonia removal in WWTPs, the comammox process may disrupt other relevant processes like anammox, because nitrite (i.e., the substrate used by anammox bacteria) is removed by comammox *Nitrospira*. Efficient nitrification-denitrification in wastewater treatment systems relies on partial ammonia removal followed by the conversion of residual ammonia and nitrite to nitrogen gas by anammox bacteria. Therefore, the interaction of comammox *Nitrospira* with anammox bacteria requires further examination and the evaluation of the effect of comammox-anammox interaction on nitrification performance, stability, and efficiency must be considered.

Although many studies have quantified nitrifying microorganisms in WWTPs, relatively few studies have attempted to assess the relative contributions of various groups to ammonia oxidation. Studies that have assessed activity typically either utilize incorporation of labelled inorganic carbon in association with ammonia oxidation or use differential inhibitors of ammonia oxidation in AOA and AOB. In one such study, Pan et al. (2018b) used ^13^C labelled substrate to show that AOA, and not AOB nor comammox *Nitrospira*, dominate ammonia oxidation in a full-scale WWTP during the winter season. Conversely, Mussmann et al. (2011) detected AOA in industrial WWTPs treating oil refinery waste but showed that these archaea were not mediating chemolithoautotrophic ammonia oxidation, despite actively expressing *amoA* genes, which was supported by a biofilm incubation experiment with similar AOA (Sauder et al., 2017. These results challenge assumptions that AOA and AOB contributions to ammonia oxidation correspond to the detection of their respective *amoA* genes. In contrast, several studies found that, regardless of whether AOA or AOB *amoA* genes dominate, both AOA and AOB assimilate labelled organic carbon (Pan et al., 2018a; Roy et al., 2017; Srithep et al., 2018), and the use of differential inhibitors has offered additional support demonstrating that both AOA and AOB can contribute to ammonia oxidation (Srithep et al., 2018). However, others showed that AOB assimilated more labelled carbon than AOA, even though AOA outnumbered AOB in the activated sludge samples (Pan et al., 2018a).

Understanding parameters relevant for shaping nitrifying microbial communities is essential for improving nitrogen removal strategies in wastewater treatment systems. Although AOB (and potentially comammox *Nitrospira*) account for most nitrification activity in municipal wastewater treatment plants, it is still valuable to explore the role that AOA and comammox *Nitrospira* play in engineered aquatic systems. To this end, contribution to nitrification cannot be inferred solely by measured AOA or AOB abundances, and it is therefore crucial to consider *in situ* activity of AOA and comammox *Nitrospira* in ecological surveys of wastewater treatment systems.

Both AOA and comammox *Nitrospira* may play additional as-yet-unknown roles in wastewater treatment systems, such as the biotransformation of compounds other than ammonia. For example, *Nitrososphaera gargensis* and *Nitrospira* species have been shown to transform the pharmaceuticals mianserin, ranitidine, asulam, and fenhexamid in co-metabolism with ammonia oxidation (Han et al., 2019; Li et al., 2022; Men et al., 2016). In addition, preliminary evidence suggests that AOA affiliated with the *Nitrosocosmicus* genus may have the potential for mixotrophic growth (Mussmann et al., 2011; Sauder et al., 2017). Based on genetic evidence, it appears that several comammox *Nitrospira* strains have the ability to use alternate nitrogen sources, such as cyanate and urea. Recently, two comammox MAGs from the RBCs and one comammox MAG from a full scale WWTP were shown to encode cyanate hydratase (the marker gene for cyanate degradation), suggesting the potential ability of comammox *Nitrospira* to grow on cyanate in ammonia-limited environments (Spasov et al., 2020; Yang et al., 2020). Several comammox *Nitrospira* MAGs also recovered in Spasov et al. (2020) contained full sets of *ure* genes, indicating the ability to hydrolyze urea to ammonia. The capability of urea hydrolysis is common amongst canonical NOB, including *Nitrospira*, and is thought to be associated with a reciprocal feeding lifestyle between strict NOB who are able to supply ammonia to ammonia oxidizers in exchange for nitrite production (Koch et al., 2015; Ding et al., 2021). Along with comammox *Nitrospira*, several AOA possess the capability to hydrolyze urea in WWTP environments, providing them with alternative nitrogen energy sources (Yang et al., 2021). Given their varying abundances in WWTPs and their potential metabolic versatility, continued research on the distributions and *in situ* activity of AOA and comammox *Nitrospira* will help determine contributions to nitrification in WWTPs.

## AOA and comammox *Nitrospira* in drinking water systems

Ammonia removal is important for drinking water systems because of strict regulatory guidelines for drinking water (Tatari et al., 2017b). AOA have been detected in several nitrifying engineered biofilter environments related to drinking water treatment, including granular activated carbon of drinking water treatment plants (DWTPs) (Kasuga et al., 2010) and groundwater treatment systems for drinking water production (Albers et al., 2015; de Vet et al., 2009, 2011; Nagymáté et al., 2016; van der Wielen et al., 2009). van der Wielen et al. (2009) used qPCR and sequencing to examine the distribution and the abundance of AOB and AOA in three groundwater source drinking water treatment plants. In this system, AOA dominated certain stages of the drinking water systems, whereas AOB dominate other stages. The overall abundance of AOA and AOB correlated positively with ammonia removal and either negatively or positively associated with dissolved organic carbon concentrations in the treatment trains, respectively. These results suggest that a high dissolved organic carbon concentrations in the water might inhibit the growth of AOA (van der Wielen et al., 2009). More recently, Poghosyan et al. (2020) used metagenomics to examine a rapid sand filter biofilm in a Dutch drinking water system and found that an AOA MAG (belonging to the genus *Nitrosoarchaeum*) constituted 0.2% of the overall assembled reads (Poghosyan et al., 2020). In drinking water purification plants from Tokyo (Japan), activated carbon filters contained both AOA and AOB, and suggested that ozonation may govern AOA populations in the filters (Niu et al., 2016). Furthermore, AOA *amoA* genes dominated over those of AOB within household sand filters (Nitzsche et al., 2015). In contrast, in rapid sand filters treating groundwater, AOA were at a low abundance compared to AOB (Fowler et al., 2018; Palomo et al., 2016; Roy et al., 2020; Tatari et al., 2017b). The authors suggest that the length of backwashing of the activated carbon filters and ammonia concentrations may play roles in niche differentiation.

Many drinking water systems use chloramine for disinfection, which releases ammonia as it decomposes. AOA have been detected in drinking water distribution systems, where nitrifying microorganisms are often considered a nuisance because they accelerate the decomposition of chloramine, which decreases the residual disinfectant (Regan et al., 2002; Wang et al., 2017). The growth of strict ammonia oxidizers may also pose a problem because of the production of nitrite, which is also toxic (Lytle et al., 2007). Archaeal *amoA* genes were detected in two drinking water distribution systems in Ontario, Canada (Scott et al., 2015), and their presence was stable over the course of a nine-month sampling period. In addition, AOA were detected in biofilms of simulated premise plumbing systems for drinking water delivery, using both copper and PVC as pipe materials (Santillana et al., 2016). In these actively nitrifying systems, archaea were detected by fluorescence *in situ* hybridization (FISH) and PCR, and associated gene sequences clustered with the *Nitrosotenuis* cluster within Group I.1a.

Since their discovery, comammox *amoA* sequences have been found in metagenomes of drinking water distribution systems and groundwater wells (Daims et al., 2015). Using metagenomic sequence data from a drinking water plant, Pinto et al. (2015) identified *amoABC* and *hao* genes on the same scaffold in a bin that belonged to the *Nitrospira* genus. The *amoA* sequence in this genome bin clustered with the *amoA* genes of comammox *Nitrospira* cultures. Another PCR-based study revealed three drinking water treatment plants and one groundwater well that contained *amoA* genes of both clades of comammox *Nitrospira* (Pjevac et al., 2017). In the groundwater well, comammox *Nitrospira* were more abundant than AOA or AOB, although this was not observed in one of the drinking water treatment plant samples (Gülay et al., 2019; Pjevac et al., 2017).

Several *Nitrospira* species have been previously detected in high abundance in drinking water systems, including several rapid sand filter systems (Albers et al., 2015; Fowler et al., 2018; Palomo et al., 2016). In groundwater-fed rapid sand filters from 12 full scale DWTPs, clade B comammox *Nitrospira* were the most abundant group of ammonia oxidizers present, exceeding the abundance of both AOA and AOB (Fowler et al., 2018). However, the role of comammox *Nitrospira* in this system remains unclear and further research is required to determine their activity in rapid sand filters. In another study, four comammox *Nitrospira* MAGs were retrieved from household tap-water filters from Singapore, China, and the USA, demonstrating that these organisms are widely distributed in drinking water distribution systems (Wang et al., 2017). These authors also looked for nitrifiers in other drinking water datasets, in addition to their tap water samples, and found evidence of AOA, AOB, and comammox *Nitrospira,* with *Nitrospira* frequently outnumbering other ammonia-oxidizing groups. Taken together, these studies point to a high abundance of comammox *Nitrospira* in drinking water systems, with much of the research focus on rapid sand filters. These drinking water related environments are typically low in ammonia and nitrite, and the rapid sand filters offer a place for biofilm growth, consistent with the predicted niche of comammox *Nitrospira* (Costa et al., 2006). Overall, the abundances of AOA vary greatly across different types and stages of drinking water systems and it is also unclear the extent to which AOA contribute to ammonia oxidation. Further work is needed to determine their activity and relative contribution to nitrification in DWTPs.

## AOA and comammox *Nitrospira* in aquarium biofilters and aquaculture operations

Ammonia is a metabolic waste product excreted by fish and other aquatic organisms. Ammonia toxicity is of particular concern for relatively closed ecosystems, such as aquaculture operations and home and commercial aquaria, where ammonia can accumulate quickly to lethal concentrations in the absence of active nitrification. The un-ionized form of ammonia (NH_3_) is particularly toxic to fish, with chronic stress and disease associated with concentrations exceeding 0.1 mg L^−1^ in aquarium and aquaculture systems (Andrews et al., 1988; Parker, 2002). In order to convert toxic ammonia to nitrate, aquarium biofilters are designed to promote the growth of nitrifying populations using the high surface area of filter support material (e.g., sponge, ceramic, or polymer) and rapid flow rates of aerated water. Despite their importance to fish health within many industrial biofilters, including aquaculture, little is known of the microorganisms catalyzing nitrification in association with aquarium biofilter support material.

Traditionally, AOB such as *Nitrosomonas* spp., were thought to be solely responsible for ammonia oxidation in aquatic environments, including aquaria. The use of AOB-containing aquarium supplements is widespread for promoting aquarium nitrification. Indeed, evidence suggests that AOB may play a role in aquarium nitrification. For example, Hovanec and DeLong (1996) found that *Nitrosomonas*-like bacteria from the *Betaproteobacteria* were associated with the saltwater aquaria in their study, although they only detected AOB in 2 of 38 freshwater aquaria, despite observing vigorous nitrification rates. This led the authors to conclude that an unknown group of microorganisms was performing ammonia-oxidation in freshwater aquarium biofilters. Subsequent studies determined that *Nitrosomonas* spp. could be enriched from freshwater aquarium biofilters (Burrell et al., 2001) indicating that they may exist in these environments at abundances too low for detection.

Since the discovery of AOA, several studies have investigated AOA in aquarium biofilters. The first isolated AOA representative, *Nitrosopumilus maritimus* SCM1, was obtained from sediment of a marine aquarium (Könneke et al., 2005). The *N. maritimus* genome shares high synteny with contigs obtained from the open ocean (Walker et al., 2010), demonstrating that this representative from an aquarium may be an important representative of AOA in natural environments. Furthermore, *Ca.* Nitrosotenuis aquarius, cultivated from a freshwater aquarium biofilter, demonstrates *in situ* ammonia oxidation activity, indicating that AOA contribute to ammonia oxidation in aquarium biofilters (Sauder et al., 2018). Other studies have analyzed the diversity of AOA in aquarium biofilters. Urakawa et al. (2008) identified the presence of *amoA* genes from AOB and AOA in marine aquarium biofilters from a public aquarium in Japan and suggested that the diversity of AOA and AOB decreased in low temperature marine aquaria. These studies highlight the overlooked importance of AOA in aquaria biofilters.

Additional studies have detected AOA in marine and freshwater aquaria, and have provided evidence for the numerical dominance of AOA in freshwater aquarium biofilters (Bagchi et al., 2014; Bik et al., 2019; Sauder et al., 2011). Sauder and colleagues examined saltwater and freshwater aquaria, and highlighted that most AOA *amoA* sequences derived from freshwater and saltwater cluster separately, suggesting niche adaptation of AOA to saltwater or freshwater environments. Additionally, the ratio of AOB to AOA appeared to be largely governed by aquarium ammonia concentrations (Sauder et al., 2011). However, due to their recent discovery, comammox *Nitrospira* were not considered in any of these studies. A recent survey of aquarium biofilters discovered that both comammox and AOA are dominant ammonia oxidizers within freshwater biofilters (McKnight and Neufeld, 2021). Clade A comammox *Nitrospira* were detected in all 38 freshwater biofilters sampled during the survey, indicating the prevalence of comammox *Nitrospira* in this environment was previously overlooked prior to their discovery. In contrast, there were no comammox *Nitrospira* detected in the saltwater aquaria where their biofilters were either dominated by AOA or AOB. Although the ubiquitous presence of comammox *Nitrospira* across freshwater biofilters in this survey suggests an important role in aquarium nitrification, further research is needed to confirm their activity *in situ*.

Recirculating aquaculture system (RAS) biofilters use similar strategies to WWTPs to regulate water quality and share many characteristics with aquaria. Several studies have examined ammonia-oxidizing microbial communities in aquaculture systems. In all compartments and in sampled tank water of a marine shrimp RAS, AOA *amoA* genes outnumbered bacterial *amoA* genes by orders of magnitude and were phylogenetically related to *N. maritimus* (Brown et al., 2013). Similarly, the abundance of archaeal *amoA* genes was 12-fold higher than those of bacterial *amoA* in a freshwater RAS (Khangembam et al., 2017). In addition to AOA, recent studies have identified the presence of comammox *Nitrospira* within RAS biofilters. One study reported that nitrite-oxidizing *Nitrospira* and comammox *Nitrospira* co-exist in a RAS filter at similar abundances (Bartelme et al., 2017). In the filter, comammox *Nitrospira* were approximately two-fold more abundant than AOA, and AOA were, in turn, more abundant than *Nitrosomonas* AOB. Because *Nitrosomonas* and *Nitrobacter* are commonly used to model RAS capacities, existing models to determine capacities may be inaccurate given their exclusion of abundant nitrifiers.

Similar to RAS biofilters, aquaponics systems also implement a recirculating system that combines both fish and vegetable growth, with potential roles for nitrifying microbial guilds. Comammox *Nitrospira* have recently been identified as the dominant ammonia oxidizers within a backyard aquaponics system, based on 16S rRNA gene sequencing and metagenomic data (Heise et al., 2021). The system operated at a low steady-state ammonia concentration, which corresponds well to the low-ammonia predicted niche of comammox *Nitrospira.* Interestingly, the presence of traditional AOB and AOA was negligible, which suggested that the *Nitrospira* present were responsible for nitrification in the sampled sediments (Heise et al., 2021). However, the study did not assess microbial communities present in the grow beds of the system, where nitrification also occurs.

Given the low ammonia concentrations imposed by design constraints and the predicted niche of nitrifying guilds, it is unsurprising that aquaculture systems can favor AOA and comammox *Nitrospira* over AOB (Bartelme et al., 2019). Overall, an improved knowledge of these nitrifiers offers an opportunity to fine-tune these systems by providing optimal conditions for the growth of the microorganisms that can achieve the desired nitrification rate to optimize animal production.

## Future research

Nitrification is a critical process in WWTPs, drinking water systems, and aquaculture operations, but understanding of the microbial contributions and controls on the process remains limited. AOA and comammox *Nitrospira* have frequently been detected in engineered water treatment systems but analyses typically focus on the relative abundances of nitrifiers without quantifying the contributions of various groups to nitrification. Indeed, in some WWTPs, even the basic metabolism of AOA remains unknown, given that abundant and actively growing *amoA*-encoding archaea were not performing ammonia oxidation (Mussman et al., 2011). Determination of relative contributions from various microbial groups to nitrification in the environment remains a challenge, but use of differential inhibitors, stable isotope probing, and creative FISH strategies can help further address these questions.

To assess the contributions of AOA and AOB to ammonia oxidation in environmental samples, differential inhibitors have been used, and have the potential to be applied for comammox *Nitrospira*. In addition to inhibiting AOB, allylthiourea (ATU) has also been shown to inhibit ammonia oxidation in comammox *Nitrospira* (van Kessel et al., 2015, unpublished data), which is expected given the similarity of ammonia oxidation enzymes between these groups. However, because ATU will inhibit both AOB and comammox *Nitrospira*, the contributions of each cannot be separated if they are both present in the same environment, such as in incubations of WWTP biofilm that demonstrated strong inhibition by ATU (Sauder et al., 2017). It is important to note that because ATU is a copper-chelator, it also inhibits AOA, in addition to AOB and comammox *Nitrospira*, albeit at a higher concentration compared to AOB and comammox *Nitrospira*. PTIO, a nitric oxide scavenger, has previously been shown to inhibit AOA and *N. inopinata*. In the RBCs, ammonia oxidation was also partially inhibited by PTIO, indicating that the AOA or comammox *Nitrospira* may contribute to ammonia oxidation in the RBCs. Additionally, *Nitrospira* were labelled with ^14^C-bicarbonate during incubations with ammonia. This labelling, along with the inconsistencies in activity assessments with the differential inhibitors, could be attributed to comammox *Nitrospira*, as suggested by Sauder et al. (2017). Finding inhibitors that will selectively inhibit ammonia oxidation by discrete nitrifying groups is an important step towards quantifying their distinct contributions under environmentally relevant conditions. Recent research suggests that simvastatin is a selective inhibitor of ammonia oxidation by AOA, which may be used to discern ammonia oxidation activity by AOA and AOB (Zhao et al., 2020). Currently there are no known comammox-specific inhibitors, although chlorate has been proposed as an indicator of comammox *Nitrospira* activity (Tatari et al., 2017a). The mechanism of inhibition by chlorate is thought to be due to the conversion of chlorate to chlorite by the activity of nitrite oxidoreductase and that chlorite is the true inhibitor of nitrite oxidation (Hynes and Knowles, 1983). Future research should assess its effectiveness for use as a general inhibitor of comammox *Nitrospira*. To find new nitrification inhibitors that are more selective of their target group, it would be important to study key differences in ammonia oxidation pathways between AOA, AOB, and comammox *Nitrospira*. Additionally, it would be useful to examine other compounds that target physiological differences between nitrifying groups (*e.g*., antibiotics, copper dependency, organic carbon compounds).

Stable isotope probing (SIP) is a powerful method that has been used to explore the activity of ammonia oxidizers within complex microbial communities. However, a major limitation of this method is cross-feeding, which may obscure the identity of the primary consumers of the labelled substrate. However, the recent development of a flow-through SIP technique (Flow-SIP) minimizes the occurrence of cross feeding by placing microbial cells on a membrane filter while continuously flowing labelled substrate across the membrane, resulting in the continual removal of labelled secondary metabolites (Mooshammer et al., 2021). Using this technique, the primary nitrifiers in an activated sludge sample were determined, and the authors suggest that Flow-SIP incubations with ^13^C-labelled bicarbonate and unlabeled ammonia, in conjunction with DNA- or RNA-SIP, would make it possible to distinguish between comammox and canonical NOB *Nitrospira* present in a microbial community because the NOB *Nitrospira* would not be active and labelled under these conditions (Mooshammer et al., 2021). This method holds promise in distinguishing the activity of various nitrifying groups within a given sample and doing so while allowing for exposure of sustained *in situ* conditions that reflect environmentally relevant substrate and product concentrations.

The FISH method has been used in the study of nitrifying communities and has allowed researchers to explore the spatial dynamics of nitrifiers within a biofilm environment (Matsumoto et al., 2010; Okabe et al., 1999; Suarez et al., 2019). Beyond the challenge of differentiating activity of various groups of nitrifiers, distinguishing canonical NOB-*Nitrospira* from comammox *Nitrospira* presents additional challenges given their phylogenetic similarity. Recent development of an activity-based labelling method for the detection of ammonia oxidizers may help overcome the issue of comammox *Nitrospira* identification with FISH while identifying active ammonia oxidizers in a microbial community. A novel activity-based protein profiling (ABPP) protocol enables the fluorescent tagging of cells containing catalytically active ammonia monooxygenase enzymes allowing for the identification of active ammonia oxidizers within a microbial community (Sakoula et al., 2021b). Although the ABPP was successfully shown to label active AOB and comammox *Nitrospira*, this current protocol was not successful in labelling AOA cells. When combining the ABPP with *Nitrospira* 16S rRNA targeted FISH for phylogenetic identification, comammox *Nitrospira* can be distinguished from NOB *Nitrospira* because only active comammox *Nitrospira* will be labelled through both FISH and ABPP approaches. In future studies, these methods may enable further insight into the nitrification activity of different microbial groups in water treatment systems.

To fully explore the ecophysiology, cellular kinetics, and metabolism of AOA and comammox *Nitrospira*, and to be able to test hypotheses generated by cultivation-independent methods, further enrichment cultures (or pure cultures) are needed. Additional comammox *Nitrospira* enrichment derived from WWTPs and DWTPs are of specific interest for comparison in the context of biochemical characteristics and niche differentiation. Because of their slow growth rate and low biomass yields, comammox *Nitrospira* are notoriously difficult to cultivate. A combination of size-based enrichment strategies (cell sorting), continuous feeding bioreactors, and antibiotics have been used to enrich several clade A comammox *Nitrospira* species (Daims et al., 2015; Fujitani et al., 2020; Kits et al., 2017; Sakoula et al., 2021a; Takahashi et al., 2020; van Kessel, 2015; Wang et al., 2021). There is an urgent need for standardized cultivation protocols that can be reliably used to cultivate comammox *Nitrospira*. The combination of cultivation-dependent and cultivation-independent techniques are both necessary to fill the gap in our understanding of the nitrogen cycle in engineered water treatment systems.

## Conclusion

Contributions to nitrification within engineered aquatic systems must now be reconsidered in light of the discovery of comammox *Nitrospira,* particularly given that several studies have reported the presence and dominance of the genus *Nitrospira* in WWTPs (Daims et al., 2001; Gruber-Dorninger et al., 2015; Kruse et al., 2013; Yao and Peng, 2017). It will be important to determine the nitrification activity of comammox *Nitrospira* in these engineered environments, as well as that of other nitrifying groups that are frequently found in the same habitats and may exhibit complex relationships. For example, ammonia oxidizers interact directly with nitrite oxidizers, and because of this functional dependency, these groups are often found co-localized in aggregates, providing insights into their interdependence. Exploring the activity and interactions of comammox *Nitrospira* and AOA with each other and other nitrogen-cycling community members will lead to a more complete understanding of factors that control ammonia removal in engineered water treatment systems, and could provide insights that lead to improved design and performance of these systems.

## Declaration of Competing Interest

The authors declare that they have no known competing financial interests or personal relationships that could have appeared to influence the work reported in this paper.
